# System-level concentration of services for mechanically ventilated patients can mask substantial regional heterogeneity and disorganization

**DOI:** 10.1186/cc9904

**Published:** 2011-03-11

**Authors:** DJ Wallace, DC Angus, MR Rosengart, TJ Iwashyna, JM Kahn

**Affiliations:** 1 University of Pittsburgh Medical Center, Pittsburgh, PA, USA; 2 University of Michigan, Ann Arbor, MI, USA

## Introduction

In the United States, critical care bed allocation is increasing, despite a decline in the number of hospitals. This process suggests a centralization of intensive care may be occurring even without central planning. In an effort to provide more efficient healthcare, many national healthcare systems have considered deregulating and decentralizing authority but have been wary about whether nongoverned, deregulated healthcare would yield naturally centralized care as a function of market forces. We evaluated the concentration of critical care services for mechanically ventilated patients in the state of Pennsylvania over time as a model for this in a decentralized system that is undergoing concentration.

## Methods

We performed a retrospective cohort study using Pennsylvania discharge data. All adult intensive care discharges between 2004 and 2008 with procedure codes for mechanical ventilation were eligible. We examined regional population-adjusted mechanical ventilation rates and the concentration of services over time. We evaluated changes in the Herfindahl-Hirshman Index (HHI), an accepted measure of overall market concentration, with larger numbers indicating greater concentration.

## Results

Hospital numbers declined over the 4 years (180, 177, 173, 173), while the number of discharges remained constant (37,635, 36,883, 37,701, 37,793). At the state level, the annual rate of discharge did not change (3.04 per 1,000 persons in 2004 to 3.05 in 2008). However, there was substantial regional variability, with three regions increasing in volume, two decreasing, and four remaining unchanged. At the state level, services were unconcentrated and did not change over time: the HHI was 160 in 2005 and 166 in 2008; however, some regions substantially concentrated while others remained the same. The most concentrated regions in 2005 (HHIs: 1,751, 2,239 and 2,886) became more concentrated by 2008 (HHIs: 1,925, 3,532, 3,564).

## Conclusions

Left to their own devices, some regions seem to centralize while others remain stagnant. Isolation of factors that drive adaptive concentration of services could be fruitful for national health systems interested in combining deregulation with centralization. Policy is needed to support outcomes-based regionalization, as a haphazard redistribution risks falling out of step with overall public health objectives if only global control of bed allocation is used.

